# Do Less Mindful Mothers Show Better Parenting via Improvements in Trait Mindfulness Following a Military Parent Training Program?

**DOI:** 10.3389/fpsyg.2019.00909

**Published:** 2019-04-24

**Authors:** Na Zhang, Jingchen Zhang, Abigail H. Gewirtz

**Affiliations:** ^1^Department of Psychology, Arizona State University, Tempe, AZ, United States; ^2^Department of Family Social Science, University of Minnesota Twin Cities, St. Paul, MN, United States; ^3^Institute of Child Development, University of Minnesota Twin Cities, St. Paul, MN, United States

**Keywords:** behavioral parent training, parenting intervention, personalized prevention, emotion socialization, moderated mediation

## Abstract

Parental deployment to war poses risks to children's healthy adjustment. The After Deployment Adaptive Parenting Tools (ADAPT) program was developed for post-deployed military families to promote children's well-being through improving effective parenting. ADAPT combines behavior management with emotion socialization skills for parents, using brief mindfulness practices to strengthen emotion regulation. We used a three-wave longitudinal, experimental design to examine whether ADAPT improved parental trait mindfulness (PTM), and whether the effect was moderated by baseline PTM. We also investigated whether improved PTM was associated with behavioral, cognitive, and emotional aspects of parenting such as self-reported parental locus of control (PLOC), self-reported parental emotion socialization (PES), self-reported and observed behavioral parenting skills. We analyzed data from a randomized controlled trial (RCT) of the ADAPT, with a focus on mothers (*n* = 313) who were either deployed (17.9%) or non-deployed and partnered with a husband who had been recently deployed to Iraq and/or Afghanistan and returned (82.1%). Families identified a 4–13-year-old target child (Mean age = 8.34, *SD* = 2.48; 54.3% girls) and were randomized into ADAPT (a group-based 14-week program) or a control condition (services as usual). At baseline, 1-year, and 2-year follow-up, PTM, PLOC, PES, and parenting skills were self-reported, whereas home-based family interactions involving parents and the child were video-taped and assessed for observed behavioral parenting skills such as discipline and problem-solving using a theory-based coding system. Results showed that mothers with lower baseline PTM reported higher PTM at 1-year while mothers with higher baseline PTM reported lower PTM at 1-year. PTM at 1-year was associated with improved self-reported parenting skills and supportive PES at 2-year, as well as indirectly associated with improved PLOC and reduced nonsupportive PES at 2-year through PTM at 2-year. No associations between PTM and observed parenting skills were detected. We discuss the implications of these findings for incorporating mindfulness practices into behavioral parenting interventions and for personalized prevention considering parents' pre-existing levels of trait mindfulness as a predictor of intervention responsivity.

## Introduction

Since the start of the War on Terror, the lives of more than two million American children have been affected by the deployment of a parent to Iraq and Afghanistan (Department of Defense., 2016). Parental deployment is a unique family stressor that can negatively affect children's adjustment. While military children are resilient and do not necessarily show adjustment problems (Meadows et al., 2016), some evidence suggests that children of deployed parents exhibited elevated levels of risk for internalizing and externalizing behaviors (Chartrand et al., 2008; Lester et al., 2010; Pexton et al., 2018), as well as alcohol and drug use problems (Acion et al., 2013). This may be partially due to compromised parenting during stressful times including reintegration following a deployed parent's return. Parenting is a crucial protective factor for children's well-being under environmental stress, and behavioral parent training programs have shown substantial evidence in preventing child behavioral problems over the long term in at-risk samples (Sandler et al., 2011; Forehand et al., 2014). In this article, we report data drawn from a randomized controlled trial (RCT) of a parenting intervention developed for post-deployed military families. Using moderation and mediation analyses, we investigated whether less mindful mothers (i.e., those with low baseline trait mindfulness) reported improvements in trait mindfulness following intervention, at 1-year post-baseline, and whether improved trait mindfulness mediated changes in parenting outcomes at 2-years post-baseline.

## Parenting From a Behavioral Perspective

Effective parenting is defined as “a broad range of functions that parents engage in to promote their offspring's accomplishment of culturally and age appropriate developmental tasks and to reduce problem behaviors” (Sandler et al., 2011). Specifically, from a social interaction learning theory perspective (see Forgatch et al., 2004), effective parenting skills involve skill encouragement to promote competencies (e.g., using praises when the child finishes homework before bedtime), limit setting and use of control strategies to discourage problematic behaviors (e.g., taking away privileges when the child comes home too late), monitoring and supervision (e.g., being aware of the child's activities in school), and effective problem solving (e.g., scaffolding the child to solve problems). In addition, effective parenting also requires positive parent-child relationships that are nurturing for child development (e.g., being positively involved with the child).

## Parental Trait Mindfulness and Parenting

Trait mindfulness refers to individual differences in the general tendency to pay attention to the present moment non-judgmentally (Brown and Ryan, 2003). Parental trait mindfulness (PTM) may be associated with effective parenting, as suggested by a growing body of literature on this topic (Conner and White, 2014; Parent et al., 2014, 2016; Riley et al., 2018). Theoretically, PTM may be linked to better cognitive capacities, emotion regulation, and fewer psychopathological symptoms or less stress (see Tomlinson et al., 2018), which in turn may be associated with effective parenting, namely, more positive and less negative parenting (Crandall et al., 2015). Parent et al. (2016) found that PTM was indirectly and negatively associated with behavioral problems in children and adolescents through increased mindful parenting and decreased negative parenting such as intrusive and coercive parenting, hostility during parent-child interactions, and ineffective disciplines. Campbell et al. (2017) showed that PTM was positively associated with parents' acceptance, affection, and responsiveness to children's needs, and this association was mediated by reduced parenting stress.

## A Third-Wave Cognitive Behavioral Approach to Parenting Interventions

The first-, second-, and third-wave of cognitive behavioral approaches are often considered to be distinct from each other (Brown et al., 2011). While the first-wave focused on predicting and changing maladaptive behaviors, the second-wave shifted the focus to changing dysfunctional beliefs as ways to reduce negative emotions and maladaptive behaviors; the third-wave emphasizes the awareness and acceptance of inner experiences as ways to change one's relationship to suffering. While the distinction conveys important messages about the differences in mechanisms of change theorized in these models, researchers have also argued that the distinction is philosophical and theoretical rather than technological and practical (Herbert and Forman, 2013).

From a third-wave cognitive behavioral approach, researchers have tested mindfulness-based programs for parents including the Mindfulness-Based Stress Reduction program (MBSR; Kabat-Zinn, 1990) with a focus on parents' stress, mental health, or parents' inner experiences rather than behavioral parenting (Bögels and Restifo, 2014). The central focus is on teaching parents a variety of mindfulness meditation (e.g., 45 min meditation per day for 6 days per week). While participants' parenting experiences may be discussed, no behavioral parenting skills are taught. A few RCTs have evidenced the outcomes of mindfulness-based programs for parents and their children, including reduced parental stress (Chaplin et al., 2018), improved parental mental health (Dykens et al., 2014; Neece et al., 2018), as well as reduced child behavioral problems (Neece et al., 2018). Nonetheless, many studies in this area lacked experimental designs in their evaluation, as the research field is still in its infancy, and it is unclear to what degree these mindfulness-based parenting programs are effective for enhancing behavioral parenting skills.

A different approach is to incorporate mindfulness into existing evidence-based behavioral parent training programs (e.g., Dawe and Harnett, 2007; Coatsworth et al., 2010; Whittingham et al., 2016; Lengua et al., 2018). Because many behavioral parent training programs target several putative mechanisms all at once, for example, to improve parenting and at the same time to reduce barriers (e.g., mental health problems or stress) to using parenting skills (Sandler et al., 2011), there is an opportunity for the integration of mindfulness into a parenting intervention that is focused on teaching parents to use behavioral strategies in parenting. We choose to call such programs mindfulness-informed parenting interventions (e.g., Zhang et al., 2018a). Instead of focusing on meditation, these programs teach mindfulness exercises to enhance parental emotion regulation and attention, reduce reactivity, and promote compassion for the child, in addition to what is typically taught in a behavioral parent training program (e.g., relationship, management of children's behaviors). Because in each session only limited time is available for teaching mindfulness, and participants have other parenting-related assignments for their home practice, these programs often use relatively brief, low dose mindfulness exercises (e.g., 5 or 10 min). Emerging evidence suggests the promise of mindfulness-informed parenting interventions. For example, Coatsworth et al. (2015) reported findings from a three-arm randomized trial, comparing the Mindfulness-Enhanced Strengthening Families Program (MSFP 10-14) to the original Strengthening Families Program 10–14 and a control condition. Their results demonstrated benefits to incorporating mindfulness practices on improved mindful parenting, parent-child relationships, and effective monitoring among fathers (measured via parents' or youth' reports).

## After Deployment Adaptive Parenting Tools/ADAPT program

After Deployment Adaptive Parenting Tools (ADAPT) is a mindfulness-informed, web-enhanced parenting program for post-deployed military families (Gewirtz et al., 2011; Pinna et al., 2017). Based on social interaction learning theory, ADAPT retains the key components of the Parent Management Training—Oregon model (PMTO), an evidence-based behavioral parent training program developed to prevent child conduct problems (Forgatch and Gewirtz, 2018). Some major modifications of the ADAPT program (see Pinna et al., 2017) include providing low doses of mindfulness practice for parent emotion regulation and emotion socialization (which involves frequent emotion discussions, teaching the child about what emotions are and how to regulate them and express them in a way that is appropriate given the child's developmental stage; see Fabes et al., 2002). ADAPT is now available in multiple formats and dosages, but in this study we evaluated, using a RCT, a 14 session group-based format of the program. In each session, a brief mindfulness exercise is introduced and is then assigned as part of the home practices for that week. Throughout the program, a variety of mindfulness exercises (lasting between 2 and 20 min) are taught, including body scan, sitting and observing, loving kindness, and mindful yoga (for more details, see Zhang et al., 2018a). The purpose of these exercises is to enhance parental emotion regulation rather than promote mindful parenting *per se*. Previous studies have shown that ADAPT was effective in improving observed couple parenting skills and child adjustment (Gewirtz et al., 2018), parenting self-efficacy (Piehler et al., 2018), and parental emotion socialization (Zhang et al., 2018b).

To date, no studies have yet examined whether the ADAPT increased PTM and how increased PTM might relate to intervention effects on improved parenting. Zhang et al. (2018a) analyzed the parents in the intervention group, finding that mothers' engagement in online mindfulness home practices in the ADAPT was associated with increased PTM at 6-month, but overall engagement was low. It is unknown whether parents randomized into the intervention showed increased PTM relative to those assigned to the control condition, and whether increased PTM would mediate the intervention effects on parenting outcomes. Just one experimental study has conducted a mediation analysis for an outcome measure of parent-child relationship quality: Coatsworth et al. (2010) reported a pilot RCT and found that the MSFP 10-14 showed intervention effects on parent-youth relationships at post-test indirectly through changes in mindful parenting. No published intervention studies have used an experimental design to test whether PTM was responsible for improved parenting outcomes over a longer term.

## The Current Study

Given prior research indicating the benefits of mindfulness for self-regulation (Tomlinson et al., 2018), and literature showing that effective parenting requires self-regulation (Dix, 1991; Crandall et al., 2015), we were interested in whether improvement in PTM might be a mediator for improved parenting in ADAPT. We did not expect an intent-to-treat (ITT) intervention effects on PTM, because the dosage and parents' engagement were low. We expected that the improvements would vary depending on baseline PTM levels, i.e., a moderated effect. Because preventive interventions often demonstrate most of their impacts for subgroups with poorer functioning when they enter the program (Tein et al., 2004; Howe et al., 2016), our first hypothesis is that mothers with lower levels of baseline PTM would show more improvements in PTM at 1-year if they were assigned to the ADAPT. Our second hypothesis is that program induced improvements in PTM at 1-year would mediate improvements in parenting at 2-year. In the current study, we measured several aspects of parenting in mothers: self-reported and observed parenting skills, self-reported parenting self-efficacy, and self-reported parental emotion socialization (PES).

Mothers are primary caregivers, and in particular, they are more likely to be the non-deployed parents in military families. We excluded fathers from the current study based on earlier findings showing no significant main or within intervention group effects of ADAPT on fathers' PTM, as well as no significant main effects on observed fathers' parenting or emotion socialization at posttest or 1-year. These are consistent with other studies showing gender differences in benefits of trait mindfulness following interventions (Rojiani et al., 2017).

## Methods

### Sample

We analyzed data collected from 313 mothers and their families who participated in an RCT of ADAPT (see Gewirtz et al., 2018 for the detailed information on the participant composition). Most mothers were non-deployed (82.11%) but partnered with a male National Guard/Reserve service member who had been deployed to Iraq and/or Afghanistan. Of the 56 deployed mothers (17.89%), 71.43% had been deployed for a cumulative length of < 18 months and 73.21% had been deployed once. They were predominantly European Caucasian (91.37%) and non-Hispanic (93.29%), married (87.86%), and on average aged 35.69 years (range = 23.05–51.15, *SD* = 5.90). Their socio-economic status was mostly middle-to-upper class (42.8% of families reported annual household income between $40,000 and $79,999, and 30.2% between $80,000 and $119,999). Half of them reported having at least a bachelor's or higher degree (51.44%), and 39.63% attended to a community college or had an associate degree. All families had a target child in the study. The children were on average 8.39 years old (range = 4.06–13.86, *SD* = 2.52) at study entry, and about half were girls (53.6%).

### Procedures

**Figure 1:**
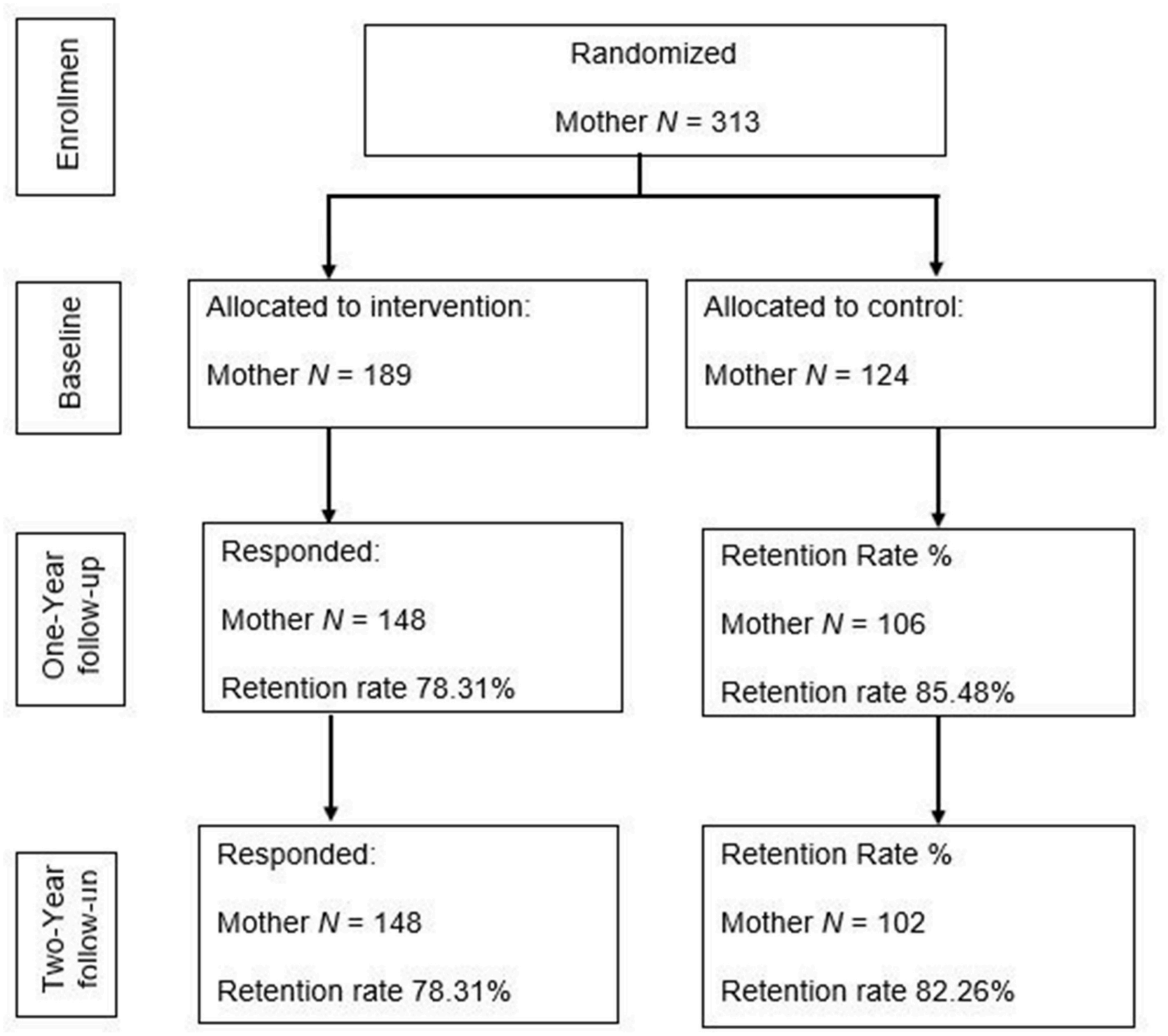
A CONSORT diagram of the current study. In the larger project, a total of 595 military families provided information on the initial screener, 259 of them was not randomized because of the following reasons: 54 families were not eligible to participate, 152 families were unable to locate or contact, and 53 families refused to participate prior to randomization. As a result, 336 families were randomized which consisted of 314 mothers and 294 fathers. One of the two mothers from a same-sex-parents family was excluded from the analyses. Anecdotally, study dropouts may be due to the stress of daily lives—work, parenting, and military service—which placed a great deal of pressure on parents and they reported not having additional time to continue in the study.

A CONSORT flowchart is shown in Figure 1. Families were eligible to participate in the study if at least one parent had been deployed to Afghanistan and/or Iraq since 2001, and at least one child was 4–13 years old. Participants were recruited using multiple strategies: presentations at military events, postings on social media, flyers, and word of mouth. Interested families completed an online survey to be screened for eligibility. Of the 336 families enrolled, 272 families had two parents participating in the study and 64 families had only one parent participating (41 mothers and 23 fathers). Families completed baseline online surveys and in-home assessment, and subsequently were randomized to the ADAPT intervention (60%) or a control condition (services as usual; 40%) (computer-generated randomization). Families in the control condition were emailed a list of “tip sheets” and online parenting resources shortly after their completion of the baseline assessment. After completing the intervention, parents received online links to surveys at 6 months as post-test. Online surveys and in-home assessments were conducted at 1-year and 2-years follow-up. Each parent received a $25 gift card for their completion of an online survey as well as a $50 gift card for the completion of an in-home assessment. All procedures were approved by the University of Minnesota's Institutional Review Board. Before the study was conducted, written informed consent was obtained from all adult participants. Children provided assent while their parent provided written consent.

### Intervention

The program consisted of 14 sessions, delivered by 2–3 trained facilitators weekly in groups of 6–15 parents. The intervention was led by two to three facilitators who were Master's level practitioners in any human services field including (but not limited to) social work, psychology, school counseling. They received extensive training prior to implementing the intervention and who received ongoing consultation throughout the study. Each session lasted about 2 h. Six parenting skills were taught, including skill encouragement, positive involvement, problem-solving, monitoring, discipline, and emotion socialization, with the first five domains rooted in the PMTO model (Gewirtz et al., 2014). Each session built on the knowledge and skills taught in the prior session, with active teaching tools including role-play and practice of skills, and discussion with other participants. Two key innovations of ADAPT, rarely addressed in prior skill-based parent training programs, were mindfulness and emotion coaching. Mindfulness was integrated into the ADAPT to enhance parental emotion regulation and to facilitate emotion coaching of children (see, Kehoe and Havighurst, 2018). Emotion coaching is a construct in the meta-emotion philosophy (Gottman et al., 1996), which suggests that parents as emotion coaches are aware of their own and their children's emotions (likely facilitated by better PTM), view negative emotions as opportunities for intimacy or teaching, can discuss emotions and help their children to understand and regulate emotions. Facilitators guided the manualized mindfulness exercises in each session, which included mindful eating, body scan, and 10 deep breaths, etc. Each exercise took 2–20 min. Participants received handouts including tips for mindfulness practice and home practice assignments. Online mindfulness exercises were also available for parents to practice outside of the group sessions.

Intervention fidelity was observed via videotapes of sessions, and coded according to facilitator knowledge, structure, teaching, process, and overall skills. Videotapes of group sessions were used to provide coaching to facilitators. Almost all content was covered (>90%). As coverage of content is core to the fidelity model, it was checked weekly in coaching sessions using the videotapes of the sessions. A total of 27 intervention groups were run, with an average size of 6–10 families per group.

Attendance and engagement data have been documented in prior reports (Doty et al., 2016; Pinna et al., 2017) and are briefly described here. Among the 207 families in the intervention group (60% of the whole sample), 156 families attended at least one group session whereas 19 families did not attend group but accessed the web-based program of ADAPT (which was designed to assist parents' engagement in the program especially if they did not attend in-person groups). Among those who attended group sessions, at least one parent in the family attended 70.66% (*SD* = 27.16%) of the total sessions on average. Families who attended group sessions completed 63.45% (*SD* = 27.86%) of the total home practice assignments.

### Measures

#### Group Assignment

Group assignment was dummy-coded as 1 = ADAPT and 0 = control condition.

#### Demographic Variables as Covariates

Mothers' deployment status (whether they had been deployed to Iraq and/or Afghanistan; 0 = nondeployed, 1 = deployed), education, age, and marital status (0 = single, 1 = married), as well as target child's age and sex (0 = boy, 1 = girl) were controlled.

#### Parental Psychological Distress and Negative Life Events as Covariates

Parental post-traumatic stress symptoms and negative life events were entered as covariates. The Post-Traumatic Stress Checklist (PCL; Weathers et al., 1993) was used to assess parents' post-traumatic stress symptoms. Deployed parents completed the military version (PCL-M), and nondeployed parents completed the civilian version (PCL-C). Each version consisted of 17 items, which were rated on a 5-point scale ranging from 1 (not at all) to 5 (extremely). The composite scores were obtained, and a dichotomous variable was then created to indicate a likely diagnosis of post-traumatic stress disorder (PTSD) status based on the clinical cutoff criteria. Parents who met the clinical cutoff criteria were coded as 1, while parents who did not meet the criteria were coded as 0. In the current sample, 6.39% percent of mothers met the criteria at baseline. The Life Events Questionnaire (LEQ; Sarason et al., 1978; Norbeck, 1984) was used to measure parents' negative life events occurring in the past year, whether each event was perceived as positive or negative, and how strong the effect was. The total counts of negative events were used in the analysis.

#### Parental Trait Mindfulness (PTM)

The Five Facet Mindfulness Questionnaire (FFMQ; Baer, 2006) was used to measure parents' trait mindfulness at baseline, 1, and 2-year follow-up. The FFMQ is a widely used instrument for assessing trait mindfulness with good internal consistency and validity (Baer et al., 2008). The scale consists of 39 items which address five dimensions of trait mindfulness: (1) observing (e.g., “When I take a shower or bath, I stay alert to the sensations of water on my body.”); (2) describing (e.g., “I'm good at finding words to describe my feelings.”); (3) acting with awareness [e.g., “I rush through activities without being really attentive to them.” (reverse coded)]; (4) non-judging of inner experience [e.g., “I make judgments about whether my thoughts are good or bad.” (reverse coded)]; and (5) non-reactivity to inner experience (e.g., “I watch my feelings without getting lost in them.”). Each item was rated on a 5-point scale (1 = “never or very rarely true,” 5 = “very often or always true”). The composite scores were used such that higher scores indicate higher levels of mindfulness. Possible range of scores for composite FFMQ is 39-195. The Cronbach's αs at baseline, T3, and T4 were 0.90, 0.92, and 0.92 for the current sample.

#### Supportive and Nonsupportive Parental Emotion Socialization (PES)

Supportive and nonsupportive parental emotion socialization (PES) practices were measured with the Coping with Children's Negative Emotions Scale (CCNES; Fabes et al., 1990) at baseline, 1, and 2-year follow-up. The CCNES is a widely used scale with adequate internal consistency and reliability (Fabes et al., 2002). Mothers were asked to endorse their responses to 12 scenarios in which children may experience negative emotions, such as fear, anger, and sadness. The scale has six subscales: emotion-focused reaction (e.g., EF; “try to make my child happy by talking about the fun things we can do with our friends”), problem-focused reaction (e.g., PF; “tell my child that the present can be exchanged for something the child wants”), expressive encouragement (e.g., EE; “encourage my child to talk about his/her fears”), minimization reaction (e.g., MR; “tell my child to quit over-reacting and being a baby”), punitive reaction (e.g., PR; “tell my child to straighten up or we'll go home right away”), and distress reaction (e.g., DR; “get upset with him/her for being so careless and then crying about it”). For each reaction under each scenario, parents responded the likelihood they would react to their children on a 7-point Likert scale (1 = very unlikely; 7 = very likely). The Cronbach's αs at baseline, 1-year, and 2-year follow-up were above 0.87 for unsupportive subscale, and the Cronbach's αs at baseline, 1-year, and 2-year follow-up were above 0.90 for supportive subscale. For this report, we conducted principle component analysis using SPSS 25.0 (IBM Corp, 2017) and created factor scores for nonsupportive PES (from PR, MR, and DR) and for supportive PES (from PF, EF, and EE).

#### Parenting Self-Efficacy (PLOC)

Parenting self-efficacy (PLOC) was measured through the Parenting Locus of Control-Short Form Revised (PLOC-SFR; Hassall et al., 2005) at baseline, 1, and 2-year follow-up. It consists of 24 items measuring four domains: parental efficacy (e.g., “I am often able to predict my child's behavior in situations”), parental responsibility (e.g., “When my child is well-behaved, it is because he/she is responding to my efforts”), child control of parents' life (e.g., “I feel like what happens in my life is mostly determined by my child”), and parental control of child's behavior (e.g., “I always feel in control when it comes to my child”). Parents were asked to rate on a 5-point Likert scale (1 = “strongly agree,” 5 = “strongly disagree”). A composite score was created with higher score indicating internal LOC, while lower score indexing external LOC. The Cronbach's αs at baseline, 1, and 2-year follow-up were 0.75, 0.76, and 0.78 in the current sample.

#### Self-Reported Parenting Skills (APQ)

The short form of Alabama Parenting Questionnaire (APQ-9; Elgar et al., 2007) was used to measure parenting skills at baseline, 1, and 2-year follow-up. The short scale has shown adequate internal consistency and criterion validity, and has linked to child disruptive behavioral problems (Elgar et al., 2007). It consists of 9 items measuring parenting skills in three domains: positive parenting (e.g., “You compliment your child after he or she has done something well”), inconsistent discipline (e.g., “Your child talks you out of being punished after he or she has done something wrong”), and poor supervision (e.g., “Your child is out with friends you don't know”). Parents were asked to rate the likelihood of each behavior on a 5-point Likert scale (1 = “never,” 5 = “always”). A composite score was created with higher score indicating more positive parenting behaviors. The Cronbach's αs were marginally acceptable in the current sample at baseline, 1, and 2-year follow-up were 0.60, 0.63, and 0.71, respectively.

#### Observed Parenting Skills (FITs)

Structured family interaction tasks (FITs) were conducted to obtain direct observations of parent-child interactions. Parents and children (father-child, mother-child, father-mother-child) were asked to complete a series of tasks, including problem-solving tasks (e.g., homework, cleaning bedrooms, bedtime, etc.), deployment-related discussions, monitoring, teaching (playing games under parents' instructions), and fun family activities. The interaction tasks lasted for approximately 40 min, and were videotaped for further coding. Observers, who were blind to the intervention conditions, coded the FITs using a Coder Impressions System (Forgatch et al., 1992), which is a macro coding system assessing both verbal and non-verbal parenting skills. The majority of the coders were undergraduate research assistants who were trained for 60 h in group training sessions led by a senior coder. Biweekly reliability meetings were held immediately following training to minimize observer drift. Twenty percent of the videos were randomized selected to assess inter-rater reliability at each time point using intraclass correlation coefficients (ICCs).

Five indicators were used to measure parenting skills: (1) problem-solving, (2) skill encouragement, (3) monitoring, (4) harsh discipline, and (5) positive involvement. The FITs scales have demonstrated adequate construct validity in prior studies (Forgatch and DeGarmo, 1999). Problem-solving was rated on a nine-item scale to evaluate the quality of the parent-child solution, the likelihood of the family putting the solution to use, extent of resolution, and the satisfaction at the discussion outcomes (α = 0.87–0.89; ICC = 0.88–0.94). Skill encouragement was rated on an eight-item scale to evaluate parent's ability to promote children's skill development through encouragement and scaffolding strategies (α = 0.76–0.83; ICC = 0.72–0.76). Monitoring was rated on a four-item scale to evaluate parents' supervision and knowledge of their child's daily activities (α = 0.60–0.71; ICC = 0.74–0.64). In these three scales, items were rate on a 5-point Liker scale from 1 to 5 (1 = “untrue,” 5 = “very true”). Harsh discipline was rated on an eight-item scale to evaluate overly strict, coercive, authoritarian, inconsistent parenting behaviors (α = 0.75; ICC = 0.58–0.78). Positive involvement was rated on a 10-item scale to evaluate parents' warmth, empathy, affection, and encouragement toward their children (α = 0.75–0.76; ICC = 0.76–0.84). Items in the last two scales were rated on a 6-point Likert scale from 1 to 6 (1 = “never,” 6 = “always”). A composite score was created among the 5 indicators with high score reflecting more effective parenting.

### Analytical Strategy

Data analyses were conducted in several stages: first, bivariate correlations were computed for key variables and *t*-tests were used to detect baseline differences on key variables between the intervention and control group. Second, in a multiple regression model, the ITT effects on PTM at 1-year follow-up were tested, and whether baseline PTM moderated the intervention effects was also tested by adding baseline PTM and an interaction effect (group assignment × baseline PTM) to the model. If the moderation effect were significant, the interaction effect would be added to mediation models in the following steps testing moderated mediation.

**Figure 2:**
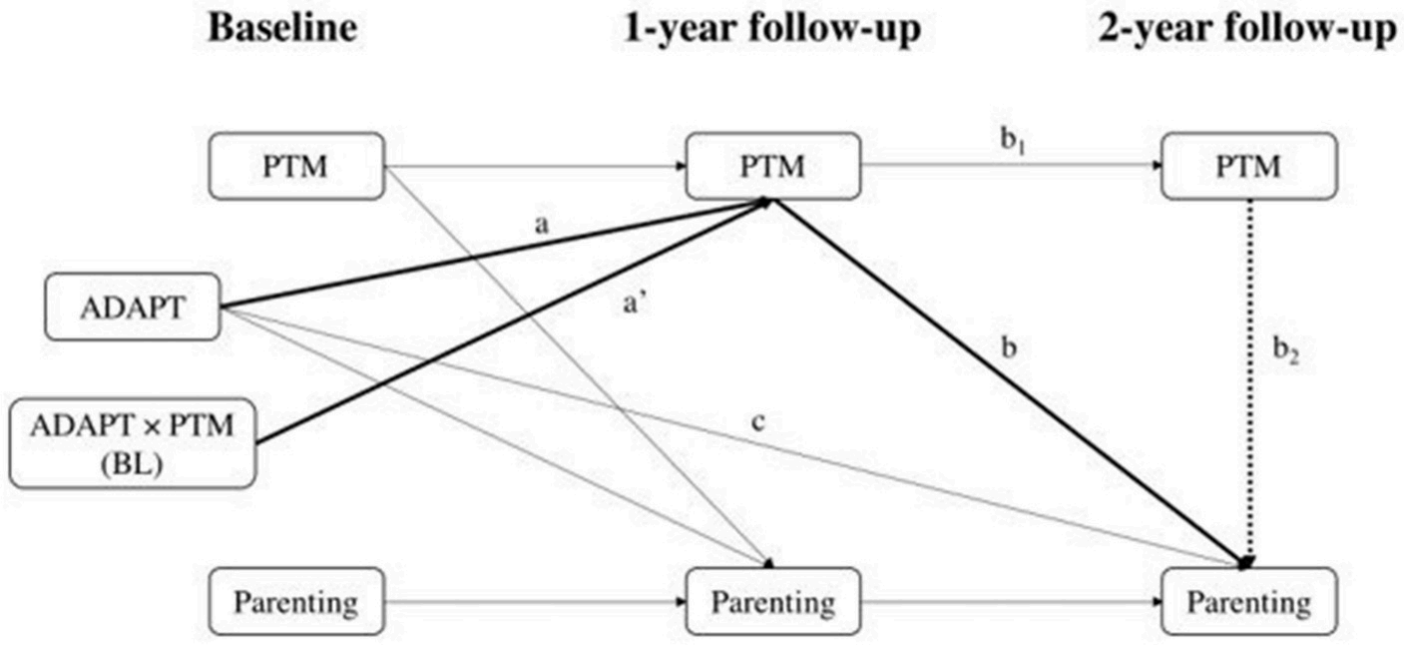
Three-wave panel models testing the mediating effects of parental trait mindfulness on parenting outcomes in the full sample. PTM, parental trait mindfulness; BL, baseline. In timely sequenced mediation models (i.e., excluding path b2), PTM and parenting are correlated at both 1-year and at 2-year, and the moderated mediating effect is ADAPT × PTM → PTM at 1-year → Parenting at 2-year (path a' and path b). In contemporaneous mediation models (with path b2), PTM and parenting are correlated at 1-year, and the moderated mediating effect is ADAPT × PTM → PTM at 1-year → PTM at 2-year → Parenting at 2-year (path a', path b1, and path b2). Covariates are not shown.

Third, we computed path models from a structural equation modeling framework to test whether improved PTM at 1-year (moderated by baseline PTM) mediated the program effects on parenting outcomes. We used path analyses with three waves of data (Figure 2) which specify the lagged correlations within each of the PTM and parenting variables across times. Such path analyses not only helps to account for the correlations between PTM and parenting both cross-sectionally and longitudinally, but they also temporally separates the measures through the time lags, which helps to reduce common method biases when all measures were self-report (Podsakoff et al., 2003). The hypothesized moderated mediation path was a' × b (*timely sequenced mediation*) or a' × b_1_ × b_2_ (*contemporaneous mediation*). Contemporaneous mediation is useful when timely sequenced mediation is not detected because of reasons such as the lagged effect of PTM on parenting does not align with the measurement times in the study (Gollob and Reichardt, 1987). To determine if there was evidence supporting a mediation effect, we used the joint significant test, which is the preferred method for hypothesis testing as it controls Type I error well and has good statistical power (MacKinnon et al., 2002; Taylor et al., 2008); there is evidence for mediation if each of the paths in the mediated effect is significantly different from zero (Taylor et al., 2008). We did not test the intervention effects on parenting outcomes because mediation effects can exist in the absence of a direct effect (Shrout and Bolger, 2002).

All models were computed in Mplus 8 (Muthén et al., 1998–2017). Model fit was evaluated using recommended criteria (McDonald and Ho, 2002), including chi-square ratio (below 2.0), comparative fit index (CFI; above 0.95), standardized root-mean-square residual (SRMR; below 0.08), and root-mean-square error of approximation (RMSEA; below 0.06). A set of covariates were included such as mothers' age, education, income, years of marriage, deployment status, PTSD status, stressful life events, child age/sex, and number of children for variables at 2-year follow-up. When model fit indices were not optimal, we removed covariates that were not significantly correlated with outcome variables and/or added a path from parenting at baseline to parenting at 2-year follow-up to improve model fit indices.

#### Missing Data

Data was missing due to reasons such as nonresponse, technical problems during in-home observation, and attrition at 1-year and 2-year follow-ups. In the current sample, the amount of missing data on variables ranged from 0 to 5.43% at baseline, 19.82–25.63% at 1-year follow-up, and 20.76~30.35% at 2-year follow-up. No demographic variables were significantly predictive of the study variable at any time point. Little's MCAR test was computed including all study variables and covariates and results supported missing at random, $\chi_{\left(573\right)}^{2}$ = 594.39, *p* > 0.05. Thus, we used Full Information Maximum Likelihood (FIML) in Mplus to handle missing data. FIML is considered less biased in comparison to other methods of dealing with missing data (Schafer and Graham, 2002).

## Results

### Preliminary Analyses

Bivariate correlations, means, and standard deviations of key variables are shown in Table 1. To summarize, PTM measures were strongly correlated across times (*rs* = 0.74–0.81). Self-reported parenting measures were moderately correlated across times (*rs* = 0.56–0.74). Observed parenting skills showed weaker correlations across times (*r*s = 0.24, 0.31, and 0.51). PTM measures were weakly-to-moderately correlated with self-reported parenting measures but not correlated with observed behavioral parenting skills.

**Table 1:** Correlations, means, and standard deviations of key variables.

**Study variables**	**1**	**2**	**3**	**4**	**5**	**6**	**7**	**8**	**9**	**10**	**11**	**12**	**13**	**14**	**15**	**16**	**17**	**18**
1. FFMQ BL	–																	
2. FFMQ 1-y	**0.76**	–																
3. FFMQ 2-y	**0.74**	**0.81**	–															
4. CCNES(sup) BL	**0.23**	**0.21**	**0.19**	–														
5. CCNES(sup) 1-y	**0.20**	0.09	**0.18**	**0.65**	–													
6. CCNES(sup) 2-y	**0.25**	**0.31**	**0.29**	**0.58**	**0.67**	–												
7. CCNES(non) BL	**−0.21**	**−0.16**	**−0.20**	**−0.18**	−0.12	**−0.14**	–											
8. CCNES(non) 1-y	−0.12	−0.09	−0.12	−0.10	−0.11	−0.05	**0.65**	–										
9. CCNES(non) 2-y	**−0.14**	−0.13	**−0.24**	−0.10	−0.06	**−0.14**	**0.63**	**0.69**	–									
10. APQ BL	**0.31**	**0.28**	**0.24**	**0.16**	**0.18**	**0.27**	**−0.21**	**−0.16**	**−0.17**	–								
11. APQ 1-y	**0.28**	**0.38**	**0.32**	**0.21**	0.13	**0.23**	**−0.32**	**−0.26**	**−0.24**	**0.62**	–							
12. APQ 2-y	**0.25**	**0.35**	**0.37**	**0.21**	**0.17**	**0.28**	**−0.26**	**−0.17**	**−0.28**	**0.55**	**0.64**	–						
13. PLOC BL	**0.29**	**0.31**	**0.31**	**0.18**	0.04	**0.22**	**−0.22**	**−0.15**	−0.12	**0.32**	**0.35**	**0.36**	–					
14. PLOC 1-y	**0.34**	**0.43**	**0.38**	**0.25**	**0.21**	**0.35**	**−0.17**	**−0.23**	**−0.18**	**0.29**	**0.37**	**0.32**	**0.66**	–				
15. PLOC 2-y	**0.24**	**0.34**	**0.35**	**0.23**	0.14	**0.36**	**−0.20**	**−0.15**	**−0.26**	**0.27**	**0.37**	**0.39**	**0.63**	**0.74**	–			
16. FITS BL	0.11	−0.01	0.01	−0.06	0.03	−0.01	**−0.16**	−0.01	−0.05	**0.12**	0.04	0.02	0.05	−0.05	−0.03	–		
17. FITS 1-y	0.05	0.05	0.04	−0.00	0.13	0.11	−0.11	−0.12	**−0.16**	**0.19**	**0.14**	0.09	0.02	0.09	0.00	**0.31**	–	
18. FITS 2-y	0.12	0.07	0.06	0.03	0.11	**0.19**	**−0.15**	−0.11	**−0.16**	0.05	0.08	0.03	−0.04	0.05	0.09	**0.24**	**0.51**	–
M	132.31	134.20	134.79	0.00	0.00	0.00	0.00	0.00	0.00	38.16	38.65	38.26	3.62	3.71	3.71	2.40	2.53	2.49
SD	17.92	17.77	18.26	0.90	0.90	0.94	0.96	0.92	0.94	3.25	3.48	3.38	0.42	0.42	0.43	0.42	0.38	0.34
Min	89.00	89.00	83.00	3.27	3.85	3.42	1.27	1.42	1.21	28.00	22.00	27.00	2.59	2.54	2.67	1.19	1.39	1.30
Max	181.00	176.00	185.00	6.82	6.82	6.82	4.33	4.24	5.09	45.00	45.00	45.00	4.76	4.79	4.92	3.31	3.61	3.29

*BL, baseline; 1-y, 1-year follow-up; 2-y, 2-year follow-up; FFMQ, Five Facets Mindfulness Questionnaire; CCNES(sup), Supportive subscale of Coping with Children's Negative Emotions Scale; CCNES(non), Nonsupportive subscale of Coping with Children's Negative Emotions Scale; APQ, Alabama Parenting Questionnaire; PLOC, Parental Locus of Control; FITS, family interaction tasks (for observed parenting skills). Bolded correlation coefficients are statistically significant, Alpha = 0.05*.

Results from independent *t*-tests showed that there were no significant differences detected on demographic variables, baseline FFMQ, PLOC, non-supportive PES, and FITS between the intervention and the control group. There was statistically significant difference on baseline APQ, *t* = 2.11, *df* = 305, *p* < 0.05, such that mothers in the intervention group had significantly higher levels of APQ (i.e., better parenting skills) than those in the control group. There was also statistically significant difference on supportive PES, *t* = 2.42, *df* = 266, *p* < 0.05, such that mothers in the intervention reported higher levels of supportive PES than those in the control group.

### Moderated Intervention Effects on PTM

**Figure 3:**
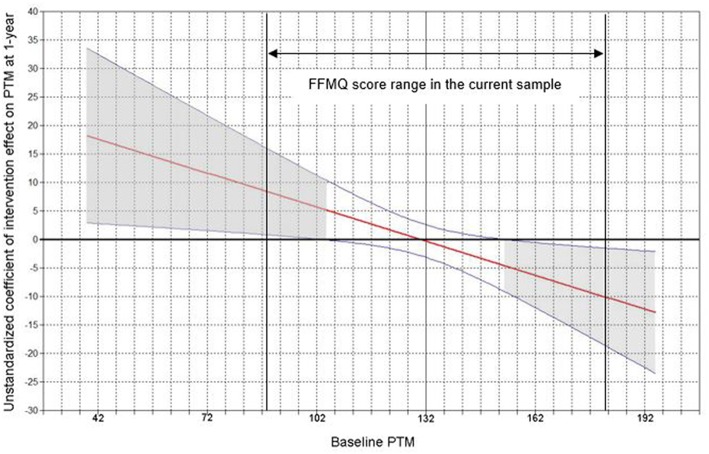
Plot of mothers' baseline trait mindfulness as a moderator conditioning the unstandardized effect of intervention on trait mindfulness at 1-year. PTM, parental trait mindfulness. The x axis is mothers' FFMQ score at baseline (possible range: 39–195; current sample: 89–181). The y axis is the unstandardized coefficient of intervention effect (effect sizes) on mothers' trait mindfulness at 1-year. Blue curved lines are 95% Confidence Intervals. Horizontal line denotes intervention effect of 0. Regions with gray shades are of statistical significance.

In a multiple regression model, the ITT effects on improved PTM were tested at 1-year follow-up, controlling for covariates as well as baseline PTM. Control variables were mothers' age, education, annual household income, years of marriage, and deployment status (1 = deployed; 0 = non-deployed). Consistent with our expectations, no significant ITT effects were found for PTM at 1-year. After baseline PTM and the interaction effect (baseline PTM by intervention) were added to the model, there was a statistically significant moderation effect (*B* = −0.20, *SE* = 0.08, β = −0.16, *p* < 0.05). Consistent with the hypothesis, region of significance (Figure 3) showed that mothers with lower levels of baseline PTM reported significantly higher PTM at 1-year if they were randomized into the intervention vs. control condition. On the other hand, a subgroup of mothers with higher levels of baseline PTM reported significantly lower PTM at 1-year if they were randomized into the intervention vs. control condition. Specifically, mothers who scored lower than 103 on the FFMQ (Z = −1.63 in the current sample; Z = −0.72 ~ −0.31 in a typical community sample, Goldberg et al., 2016) pre-intervention showed significant improvements in PTM at 1-year if they were randomized into the intervention; those who scored higher than 154 on the FFMQ (Z = 1.21 in the current sample; Z = 2.81~3.34 in a typical community sample; Goldberg et al., 2016) pre-intervention showed significantly lowered PTM at 1-year if they were randomized into the intervention; and, finally, mothers whose FFMQ scores were about the mean levels of the sample, either in the intervention group or the control condition, did not show significant changes at 1-year.

Because there was a negative intervention effect for a subgroup of mothers, we conducted *post hoc* analysis to test whether intervention effects were maintained to 2-year follow-up. Results showed that no individuals in the current sample fell into the region of significance for positive or negative intervention effects for PTM at 2-year follow-up (all *p*s > 0.05), suggesting that the impacts of the intervention on PTM at 1-year for the subgroup of mothers with lower or higher baseline PTM disappeared at 2-year follow-up.

### Moderated Mediation Effects on Parenting Outcomes

Given a significant moderated intervention effect on PTM at 1-year, we tested whether this moderated effect mediated the intervention effects on parenting outcomes. A total of five moderated mediation models were specified for PTM and each parenting outcome (Figure 2). In all models, baseline PTM consistently moderated the intervention effect on PTM at 1-year (a' path) in Figure 2, *p*s < 0.05. Below, timely sequenced mediation effect of PTM on parenting was described first, followed by contemporaneous mediation effect.

#### Self-Reported Parenting Skills (APQ)

A timely sequenced mediation model demonstrated a good fit to data: $\chi_{\left(51\right)}^{2}$ = 94.91, *p* < 0.001, χ^2^/*df* < 2.00, CFI = 0.95, RMSEA = 0.05, SRMR = 0.04. Results showed that PTM at 1-year significantly and positively predicted self-reported parenting skills at 2-years (b path) above and beyond past APQ scores and other covariates, *B* = 0.03, *SE* = 0.01, β = 0.17, *p* < 0.01. Therefore, mothers' higher PTM at 1-year was associated with better self-reported parenting skills at 2-year follow-up.

#### Supportive PES

A timely sequenced mediation model demonstrated a good fit to data: $\chi_{\left(43\right)}^{2}$ = 84.31, *p* < 0.001, χ^2^/*df* < 2.00, CFI = 0.95, RMSEA = 0.06, SRMR = 0.04. PTM at 1-year significantly and positively predicted supportive PES at 2-year follow-up (b path) above and beyond past supportive PES scores and other covariates, *B* = 0.009, *SE* = 0.003, β = 0.16, *p* < 0.01. Therefore, mothers' higher PTM at 1-year was associated with higher self-reported supportive PES at 2-year follow-up.

#### Non-supportive PES

A timely sequenced mediation model was computed. The model demonstrated a good fit to data: $\chi_{\left(53\right)}^{2}$ = 88.98, *p* < 0.001, χ^2^/*df* < 2.00, CFI = 0.96, RMSEA = 0.05, SRMR = 0.03. This model showed no significant mediated effect from PTM at 1-year to non-supportive PES at 2-year (i.e., b path was not significantly different than zero). A contemporaneous mediation model was then computed, which demonstrated a good fit to data: $\chi_{\left(54\right)}^{2}$ = 90.98, *p* < 0.001, χ^2^/*df* < 2.00, CFI = 0.95, RMSEA = 0.05, SRMR = 0.03. This model showed that PTM at 1-year strongly predicted PTM at 2-year (b_1_ path), *B* = 0.84, *SE* = 0.04, β = 0.82, *p* < 0.001, and PTM at 2-year was significantly and negatively associated with nonsupportive PES at 2-year (b_2_ path) above and beyond past nonsupportive PES scores and other covariates, *B* = −0.006, *SE* = 0.002, β = −0.11, *p* < 0.05. Therefore, while there was no direct effect of PTM at 1-year on non-supportive PES at 2-year, PTM at 1-year was associated with decreased nonsupportive PES at 2-year through PTM at 2-year follow-up.

#### Parenting Self-Efficacy (PLOC)

A timely sequenced mediation model was computed. After adding a path from baseline PLOC to 2-year, the model demonstrated a good fit to data: $\chi_{\left(51\right)}^{2}$ = 92.87, *p* < 0.001, χ^2^/*df* < 2.00, CFI = 0.95, RMSEA = 0.05, SRMR = 0.05. This model showed no significant mediation effect from PTM at 1-year to PLOC at 2-year (i.e., b path was not significantly different than zero). A contemporaneous mediation model was then computed, which demonstrated a good fit to data: $\chi_{\left(52\right)}^{2}$ = 94.67, *p* < 0.001, χ^2^/*df* < 2.00, CFI = 0.95, RMSEA = 0.05, SRMR = 0.04. This model showed that PTM at 1-year strongly predicted PTM at 2-year (b_1_ path), *B* = 0.84, *SE* = 0.04, β = 0.81, *p* < 0.001, and PTM at 2-year was significantly and positively associated with PLOC at 2-year (b_2_ path) above and beyond past PLOC scores and other covariates, *B* = 0.002, *SE* = 0.001, β = 0.11, *p* < 0.05. Therefore, while there was no direct effect of PTM at 1-year on PLOC at 2-year follow-up, PTM at 1-year was associated with increased PLOC at 2-year through PTM at 2-year follow-up.

#### Observed Behavioral Parenting Skills

A timely sequenced mediation model was computed. The model fit indices were not optimal but acceptable: $\chi_{\left(52\right)}^{2}$ = 98.07, *p* < 0.001, χ^2^/*df* < 2.00, CFI = 0.93, RMSEA = 0.05, SRMR = 0.04. This model showed no significant mediated effect from PTM at 1-year to observed parenting at 2-year (i.e., b path was not significantly different than zero). A contemporaneous mediation model was then computed with not optimal but acceptable model fit indices: $\chi_{\left(53\right)}^{2}$ = 100.42, *p* < 0.001, χ^2^/*df* < 2.00, CFI = 0.93, RMSEA = 0.05, SRMR = 0.04. Still, this model showed no significant mediated effect from PTM at 2-year to observed parenting at 2-year (i.e., b2 path was not significantly different than zero). This was not surprising given the non-significant bivariate correlations between PTM and observed parenting measures (Table 1).

## Discussion

Our goal was to understand for whom the intervention might be more or less beneficial depending on baseline levels of PTM (moderation analyses) as well as the mediating relationship between PTM and parenting outcomes. Our analyses revealed several findings: first, while no main effects of the intervention on self-reported PTM were found, baseline PTM was a moderator for the intervention effects. Specifically, mothers with lower levels of baseline PTM reported higher PTM at 1-year follow-up if they were randomized into the intervention vs. control condition; mothers with higher levels of baseline PTM reported lower PTM at 1-year follow-up if they were randomized into the intervention vs. control condition; and mothers with average levels of baseline PTM did not report significant changes from either condition. We note, with more details below, that mothers in the current sample reported much higher PTM before the group assignment, relative to other samples we found in the literature. Second, PTM in mothers at 1 or 2-year follow-up was associated with self-reported parenting skills (APQ), PLOC, and PES at 2-year follow-up in expected directions (effect sizes were small). No associations of PTM were found with observed parenting skills. Overall, the findings supported our hypotheses regarding self-reported parenting but not observed behavioral parenting. These findings provide important information to future theorists and interventionists in the studies of a third-wave cognitive behavioral approach to parenting.

In comparison to other studies in the literature (e.g., Baer et al., 2008; Goldberg et al., 2016), the sample in the current study scored much higher on the FFMQ. Prior samples have included diverse community samples: both female and male, a larger range of age, and different socio-economic backgrounds. The current sample is less diverse as mothers were mostly in their 30s, middle-income, White, and partnered with a male military service member. There are few studies examining socio-demographic correlates of trait mindfulness, especially among parents, though intervention studies do suggest that women may be more responsive to mindfulness training than men (e.g., Rojiani et al., 2017). Further research is needed to understand socio-demographic differences in self-reported PTM.

Our finding of no main effects of the ADAPT program on mothers' PTM is consistent with a recent meta-analysis reporting that self-reported gains in trait mindfulness following a range of mindfulness-based interventions are relatively modest compared with gains in clinical outcomes (Goldberg et al., 2018). The dosage of mindfulness in the ADAPT program was much smaller than mindfulness-based interventions: the mindfulness meditation exercises delivered in each session were very short, and participant engagement in mindfulness home practice was low (just half of the intervention condition sample accessed any of the mindfulness home practices online; Zhang et al., 2018a). This is not surprising given the context of mindfulness-informed parenting interventions: parents have many competing demands on their time, and home practice was not limited to mindfulness exercises as parents also were instructed to practice behavioral parenting techniques between sessions. Singh et al. (2006, 2007), in their evaluations of a mindfulness-based parenting intervention for mothers of children with developmental disabilities, found that reductions in child behavior problems occurred after mothers engaged in mindfulness practice.

According to results of the moderation analyses, even the relatively small doses of mindfulness practices in the ADAPT program, however, *were* effective for mothers who showed deficits in PTM at baseline, i.e., whose baseline FFMQ scores were below 103, which is approximately a typical civilian community sample mean (e.g., Goldberg et al., 2016). This finding suggests that even small doses of mindfulness (just a few minutes at a time) might be beneficial in boosting PTM for mothers with PTM deficits (i.e., very low self-reported observing, describing, acting with awareness, non-judging, and non-reactivity). Basso et al. (2019) found that a brief mindfulness-based intervention that requires participants to practice 13-min per day for 8 weeks were effective in decreasing negative emotions and enhancing cognitive capacities (e.g., attention, working memory) at post-test. For mothers who needed the most, the ADAPT intervention strengthened their PTM at 1-year. Strengthened PTM might help mothers to be present with their children, be less preoccupied with their own distress during parent-child interactions, and consistently use discipline or encouragement. Parents with higher levels of PTM may have better reflective functioning which helps parents to mentally represent and understand their children's internal experience while reflecting their own experience as parents, enabling meaningful and appropriate actions in the parenting context (Slade, 2005). Mindfulness exercises taught in the ADAPT such as “sitting and observing,” “loving kindness,” “stretching” (i.e., mindful yoga activities with children), may help increase parental reflective functioning as well as PTM by increasing awareness, non-reactivity, and interpersonal attunement with child. It would seem logical, then, that these small gains in PTM would be reflected in subsequent gains in perceived parenting efficacy and both behavioral and emotional positive parenting. While we did not track whether mothers engaged in mindfulness practices during the year after the intervention, it is important to note that practices are necessary for maintaining and/or boosting positive outcomes such as improved PTM. It may be helpful to include engagement boosters or relevant resources in a mindfulness-informed parenting intervention to assist parents in continuing their practices.

We were curious regarding the finding that mothers with very high levels of baseline PTM actually reported *decreased* PTM at 1-year if they were randomized into the intervention vs. control condition, though a *post hoc* analysis indicated that such effects disappeared at 2-years. We speculate that these findings may be related to the inherent differences between behavior management and mindfulness training approaches (Duncan et al., 2009). While parent training teaches parents to identify, evaluate, and respond to children's behaviors using reward or punishment, mindfulness practices, and principles focus on being in the present moment and allowing evaluative thoughts to pass by without clinging onto them. It is possible that highly mindful mothers, as they practiced ADAPT parenting skills, engaged in the judging that is required to respond to children's behaviors, which caused some cognitive dissonance with their mindful mindsets. This dissonance may have been resolved by moving toward what they perceived as a more reactive and interventionist stance vis a vis their children (rather than a more mindful approach), which may have resulted in perceptions of poorer parenting efficacy and skills.

While evidence exists supporting the relationship between self-reported mindful parenting and observed parenting behaviors in mothers (Duncan et al., 2015), we did not find associations between PTM and observed parenting in our sample. Further research within a group of highly mindful mothers may help to understand what was happening during and after their participation in a behavioral parenting intervention. It is possible that the lack of goodness-of-fit between parents and programs may disadvantage parents' own strengths (Singh, 2001). If that is the case, parents with very high levels of PTM may require a more tailored approach to learning parenting or a different approach to the sequencing of intervention components. For instance, interventions may start with mindfulness training (i.e., attention and compassion), and then frame skill encouragement and limit setting in a way that is integrated with parents' pre-existing strength in mindfulness. Given that the current sample scored on FFMQ much higher than other community and clinical samples in the literature, behavioral measures of trait mindfulness (e.g., breath counting; Levinson et al., 2014) instead of self-reports and/or qualitative data may be useful to further examine this issue.

On the other hand, such different findings between self-reports and observed measures of parenting are evident in the broader literature of behavioral parenting intervention (those without a mindfulness component). For instance, meta-analyses of evidence-based parenting interventions such as the Incredible Years program and the Triple P program have found significant program effects on improved self-reported parenting, but not on observed parenting (Nowak and Heinrichs, 2008; Sanders et al., 2014; Leijten et al., 2018). It is possible that observed parenting reflect some aspects of personality or psychopathology (McCabe, 2014) which are not the targets of parenting interventions. However, robust evidence including objectively measured parenting can further support the effects of evidence-based programs in addition to self-reports. Future researchers can develop new methods to measure aspects of parenting behaviors objectively that are sensitive to change. For example, instead of using structured parent-child interaction tasks, Sperling and Repetti (2018) used naturalistic observational methods in which families were recorded by two videographers on 2 week days and 2 weekend days without any prompts for particular activities or interactions.

Finally, we found evidence supporting the moderated mediation effect of PTM on all of the self-reported parenting measures, i.e., changes in PTM at 1-year as predicted by the interaction effect of intervention by baseline PTM were associated with self-reported parenting at 2-year either longitudinally or cross-sectionally through PTM at 2-year. While the mediation effects were statistically significant according to the joint significance test, the effect sizes of the associations between PTM and self-reported parenting measures were small. We note that this should not discourage future applications of this novel approach. In fact, small program effects can be meaningful in preventive intervention settings (vs. clinical settings) to reduce public health burden. Future research is warranted for a better understanding about what individual or family processes may moderate the relationship between PTM and parenting outcomes.

Several limitations of this study should be noted. First, the sample was NG/R military parents and thus our findings may not be generalizable to other military family contexts. However, the designs of the study and the findings reported here may be informative for clinicians, prevention interventionists and researchers in their work with at-risk families in the parenting field. Second, mindful parenting was not assessed in the ADAPT program. Mindful parenting may be more malleable than trait mindfulness in a parenting intervention with small doses of mindfulness practices. Third, while we discussed our findings in relation to a prior study about parents' engagement in mindfulness practices, parents' actual practices were not systematically measured in this study. Thus, we were unable to explore a dose-response relationship. Finally, the APQ consisted of only a limited number of items and the reliability was low in the current sample, which might explain the weak correlation between APQ and observed parenting.

Future research should examine different dimensions of PTM in relation to parenting. Studies have shown that distinct mindfulness facets are variably linked to depression, anxiety, and stress (e.g., Desrosiers et al., 2013). In this study, we used FFMQ composite scores to measure PTM because the FFMQ is one of the most widely-used scales for measuring trait mindfulness and it captures the multidimensional aspect of trait mindfulness. We did not hypothesize that the ADAPT program would demonstrate different intervention outcomes based on distinct mindfulness facets. Neither did our study aim to test which one of the mindfulness facets is more or less important in the context of a parent training program. These important questions are beyond the scope of this article but they warrant further consideration. Finally, future research should consider using behavioral measures of mindfulness (Levinson et al., 2014), which may be a more reliable method than self-reports.

## Ethics Statement

All procedures were approved by the University of Minnesota's Institutional Review Board. Before the study was conducted, written informed consent was obtained from all adult participants. Children provided assent while their parent provided written consent.

## Author Contributions

NZ developed the research question, conducted the analysis, and wrote the first draft of the manuscript. JZ assisted in data analysis, writing the methods and results sections and created the tables and figures. AG (PI of the ADAPT study) contributed to the development of the manuscript, the writing of the discussion section, and edited the paper. All authors read and approved the manuscript and agreed with the authorship order.

### Conflict of Interest Statement

The authors declare that the research was conducted in the absence of any commercial or financial relationships that could be construed as a potential conflict of interest.
